# Single chest drain is not inferior to double chest drain after robotic esophagectomy: a propensity score-matched analysis

**DOI:** 10.3389/fsurg.2023.1213404

**Published:** 2023-07-14

**Authors:** F. Eckert, F. Merboth, E. Giehl-Brown, J. Hasanovic, B. Müssle, V. Plodeck, T. Richter, T. Welsch, C. Kahlert, J. Fritzmann, M. Distler, J. Weitz, J. Kirchberg

**Affiliations:** ^1^ Department of Visceral, Thoracic and Vascular Surgery, University Hospital Carl Gustav Carus, Technische Universität Dresden, Dresden, Germany; ^2^ National Center for Tumor Diseases Dresden (NCT/UCC), Dresden, Germany; ^3^ German Cancer Research Center (DKFZ), Heidelberg, Germany; ^4^ Faculty of Medicine and University Hospital Carl Gustav Carus, Technische Universität Dresden, Dresden, Germany; ^5^ Helmholtz Centre Dresden - Rossendorf (HZDR), Dresden, Germany; ^6^ Department of General, Visceral and Thoracic Surgery, St. Elisabethen-Klinikum Ravensburg, Academic Teaching Hospital of the University of Ulm, Ravensburg, Germany; ^7^ Institute and Polyclinic for Diagnostic and Interventional Radiology, University Hospital Carl Gustav Carus, Technical University Dresden, Dresden, Germany; ^8^ Department of Anaesthesiology and Critical Care Medicine, University Hospital Carl Gustav Carus Dresden, Technische Universität Dresden, Dresden, Germany

**Keywords:** robotic esophagectomy, chest drain management, ERAS, RAMIE, chest tube

## Abstract

**Background:**

Chest drain management has a significant influence on postoperative recovery after robot-assisted minimally invasive esophagectomy (RAMIE). The use of chest drains increases postoperative pain by irritating intercostal nerves and hinders patients from early postoperative mobilization and recovery. To our knowledge, no study has investigated the use of two vs. one intercostal chest drains after RAMIE.

**Methods:**

This retrospective cohort study evaluated patients undergoing elective RAMIE with gastric conduit pull-up and intrathoracic anastomosis. Patients were divided into two groups according to placement of one (11/2020–08/2022) or two (08/2018–11/2020) chest drains. Propensity score matching was performed in a 1:1 ratio, and the incidences of overall and pulmonary complications, drainage-associated re-interventions, radiological diagnostics, analgesic use, and length of hospital stay were compared between single drain and double drain groups.

**Results:**

During the study period, 194 patients underwent RAMIE. Twenty-two patients were included after propensity score matching in the single and double chest drain group, respectively. Time until removal of the last chest drain [postoperative day (POD) 6.7 ± 4.4 vs. POD 9.4 ± 2.7, *p* = 0.004] and intensive care unit stay (4.2 ± 5.1 days vs. 5.3 ± 3.5 days, *p* = 0.01) were significantly shorter in the single drain group. Overall and pulmonary complications, drainage-associated events, re-interventions, number of diagnostic imaging, analgesic use, and length of hospital stay were comparable between both groups.

**Conclusion:**

This study is the first to demonstrate the safety of single intercostal chest drain use and, at least, non-inferiority to double chest drains in terms of perioperative complications after RAMIE.

## Introduction

Esophageal cancer (EC) is the sixth most common cause of cancer-related death worldwide (1). The introduction of minimally invasive esophagectomy (MIE) and especially robot-assisted minimally invasive esophagectomy (RAMIE) optimized postoperative outcomes by reducing surgical site infections and pulmonary complications (2–5). Minimally invasive techniques also led to a reduction of postoperative pain and to a rapid and enhanced mobilization of patients with shortened hospital stay without negatively impacting oncological outcomes (6).

Therefore, MIE has become part of international guideline recommendations worldwide (7, 8) and should be preferred to open esophagectomy (OE) in clinical practice according to German guidelines (5, 8, 9).

Over the last years, enhanced recovery after surgery (ERAS) programs were implemented in esophageal surgery to accelerate postoperative recovery and to further reduce perioperative morbidity, length of hospital stay (LOS), and consequently healthcare costs (10–12). Key points of ERAS are early postoperative mobilization and oral feeding and minimized use of drains and nasogastric tubes. Chest drain management is a crucial factor that has significant influence on postoperative recovery after RAMIE.

Thoracic drains severely increase postoperative pain by irritating intercostal nerves and hinder patients from early postoperative mobilization and recovery. Traditionally, two intercostal chest drains have been used to drain the right pleural cavity after esophageal resections, the inferior one to treat basal effusions, and the apical one to treat pneumothorax (13, 14).

However, in thoracic surgery, several randomized studies demonstrated that usage of a single chest drain, instead of two, is equally effective after anatomical lung resections without compromising perioperative outcomes (15–17). In contrast, evidence on optimal management of chest drains after esophagectomy is scarce (18). A recent review of 27 retrospective studies with 2,564 patients concluded that non-optimal chest drain management has the potential to negatively affect outcomes and emphasized the need for further trials to determine optimal management (18).

To our knowledge, to date, no study has investigated the use of two vs. one intercostal chest drain after RAMIE.

In view of the above, we hypothesize that one chest drain after RAMIE is safe in terms of postoperative complications and at least not inferior compared to two chest drains.

We compared a single chest drain cohort with a double chest drain cohort in terms of overall and pulmonary complications, drainage-associated re-interventions, diagnostic imaging, analgesic use, and LOS in the single drain group compared to the double drain cohort after propensity score matching (PSM) for potential confounders.

## Methods

### Study design and population

All patients who underwent fully robot-assisted esophagectomy at the Department for Visceral, Thoracic, and Vascular Surgery at the University Hospital Dresden between 31 August 2018 and 04 August 2022 were included in this retrospective analysis.

Inclusion criteria were biopsy confirmed malignant esophageal or esophagogastric junction tumor [adenocarcinoma (AC), squamous cell carcinoma, and other malignant tumors], elective fully robotic esophagectomy via an abdomino-thoracic approach (Ivor Lewis), reconstruction with gastric conduit pull-up and intrathoracic anastomosis using a circular stapler.

Exclusion criteria were benign histology, emergency operations, open or hybrid esophagectomy, cervical anastomosis, colon conduit, intrathoracic anastomosis using a linear stapler, placement of additional left-sided chest drains, and patients’ death in hospital or within the first 30 postoperative days (PODs).

The study protocol was reviewed by a local ethics committee (EK-84022022) and was performed in accordance with the Declaration of Helsinki and its later amendments. Some of the analyzed patient collectives have already been published regarding other end points (19, 20).

Data were collected retrospectively from the hospitals database and patients’ records by two independent investigators. General complications were graded according to Clavien–Dindo (21) and specific complications after esophagectomy were graded according to the Esophagectomy Complications Consensus Group (ECCG) (22).

### Surgical technique

The surgical technique of RAMIE has been described elsewhere (23).

In our department, the surgical procedure for esophageal resections was changed to RAMIE in August 2018. Initially, the standard insertion of two intercostal chest drains (24 Ch, recessus and apical via robotic trocars R1 and R3) was performed. From November 2020, we changed the routine placement of the right-sided chest drains to a single-intercostal drain (24 Ch, apical via robotic trocar R3) (Supplementary Figures S1,S2).

### Standard for chest drain management

According to the standard algorithm, chest x-ray is performed after surgery on admission to the intensive care unit and then every 2 days from the second POD onward to check dilatation of gastric conduit.

Before November 2020, the first peri-anastomotic apical chest drain was removed earliest on POD 2 independent of the daily secretion amount if there is no evidence of air leak (<100 ml/min), pneumothorax > 2.5 cm, chyle leak, purulent secretion, or bleeding. The second recessus chest drain was removed if the daily secretion was below 200 ml per 24 h and if there was still no evidence of air leak (<100 ml/min.), pneumothorax > 2.5 cm, chyle leak, purulent secretion, or bleeding followed by chest x-ray for control.

After change of drain management November 2020, the single drain was removed if the daily secretion was below 200 ml per 24 h and if there was still no evidence of air leak (<100 ml/min), pneumothorax > 2.5 cm, chyle leak, purulent secretion, or bleeding followed by chest x-ray for control.

### Standard for perioperative pain management

According to the standard algorithm, thoracic epidural catheter is placed in the absence of contraindications and in case of patient consent in every patient undergoing RAMIE in our institute. It is placed preoperatively (before induction of anesthesia) at the thoracic vertebral level 6–9. Initially, ropivacaine hydrochloride 0.2% plus 0.5 µg/ml sufentanil is administered. In the further postoperative course, only ropivacaine hydrochloride 0.2% is administered. The concomitant pain medication consisting of 1 g of metamizole alternatively 1 g paracetamol four times daily may be extended to 10 mg extended release oxycodone every 12 h and an on-demand medication of 10 mg immediate release oxycodone if the pain score is persistent >4 on the numeric rating scale (NRS). The epidural catheter running rate and dosage is checked daily by the anesthesia pain service and adjusted to the individual needs of the patient (including the increase of the basal rate to max. 8 ml/h, reducing/terminating the basal rate of continuous epidural infusion and removal of the catheter).

### Statistical analysis

Statistical analyses were performed using SPSS (version 28.0, IBM Corp., Armonk, NY, United States). Continuous variables were presented as mean ± SD or median with interquartile range. Continuous data were compared using Student’s *t*-test if the variables were normally distributed. The Mann–Whitney *U* test was used to compare continuous non-parametric variables. Categorical variables were compared using chi-square or Fisher’s exact tests. The significance level was set at *p* = 0.05.

To ensure better comparability between single and double drain cohorts, a 1:1 propensity score matching was performed. The following variables were used to calculate the propensity score using the following regression models: sex, age, body mass index (BMI), American Society of Anesthesiologists Classification (ASA), Charlson comorbidity index (CCI), neoadjuvant treatment, adjuvant treatment, histology, and pathologic Union for International Cancer Control (UICC) stage. Subsequently, the nearest neighbor method with a caliber width of 0.1 was used to find matching pairs.

## Results

### Patient characteristics

Between 2018 und 2022, 194 patients underwent Ivor Lewis esophagectomy for malignant disease at our center. Seventy-four patients met inclusion criteria for this study (Supplementary Table S1).

After propensity score matching, 22 patients per group were included into this analysis.

Patients were 61.7 ± 10.6 years old and predominantly male (*n* = 37, 84.1%).

Most patients had adenocarcinoma (*n* = 30, 68.1%) and underwent neoadjuvant therapy (*n* = 39, 88.6%). Neoadjuvant chemotherapy was administered in 24 patients (54.5%) and neoadjuvant chemoradiation in 15 patients (34.1%).

After propensity score matching; patients were comparable regarding baseline characteristics including age, BMI, CCI, ASA classiﬁcation, comorbidities, histology, neoadjuvant therapy, and UICC clinical stage within the single and double chest drain group (Table 1).

**Table 1:** Patient characteristics and histopathologic findings after PSM.

	Single drain	Double drain	*p*-value
	*n* = 22	*n* = 22	
**Age (years)**	61.55 (12.92)	61.86 (7.82)	0.92*
**Sex**			0.68^#^
Female	4 (18.2)	3 (13.6)	
Male	18 (81.8)	19 (86.4)	
**BMI (kg/m^2^)**	26.9 (3.4)	26.8 (5.2)	0.95*
**ASA**			0.75^#^
1	0	0	
2	7 (31.8)	8 (36.4)	
3	15 (68.2)	14 (63.6)	
4	0	0	
**Charlson comorbidity index (CCI)**	2.5 ± 0.74	2.36 ± 0.58	0.50*
**Histology**			0.22^#^
Adenocarcinoma	16 (72.7)	14 (63.6)	
Squamous cell carcinoma	6 (27.3)	6 (27.3)	
Others	0	2 (9.1)	
**Neoadjuvant treatment**	20 (90.9)	19 (86.4)	0.64^#^
Chemotherapy	12 (54.5)	12 (54.5)	1.0^#^
Chemoradiation	8 (36.4)	7 (31.8)	0.75^#^
**pUICC stage**			0.27^#^
1	11 (50.0)	7 (31.8)	
2	1 (4.5)	5 (22.7)	
3	8 (36.4)	9 (40.9)	
4	2 (9.1)	1 (4.5)	

* T-Test/ Mann–Whitney-U.

^#^ Chi-square/Fisher’s exact test.

### Duration and volume of chest drains

The first chest drain was removed earlier in the double drain group than in the single drain group but without significance (POD 6.68 ± 4.37 vs. POD 5.77 ± 1.8, *p* = 0.42, Table 2).

**Table 2:** Duration and volume of chest drains.

	Single drain	Double drain	*p*-value
	*n* = 22	*n* = 22	
Removal of first drain (POD)	6.68 ± 4.37	5.77 ± 1.8	0.423*
Removal of last chest drain (POD)	6.68 ± 4.37	9.41 ± 2.72	0.004*
Drainage volume POD 1–5 (ml)	2,450.00 ± 1,510.98	2,808.86 ± 1,209.66	0.39*
Total drainage volume until removal of last drain (ml)	3,075.00 ± 2,563.10	3,621.36 ± 1,592.30	0.41*

* T-Test/ Mann–Whitney-U.

**Figure 1:**
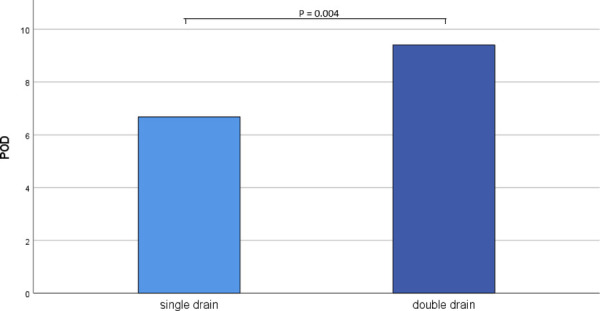
Removal of last chest drain.

In the double drain group, the final chest drain was removed significantly later than in the single drain group (POD 6.68 ± 4.37 vs. POD 9.41 ± 2.72, *p* = 0.004, Figure 1).

**Figure 2:**
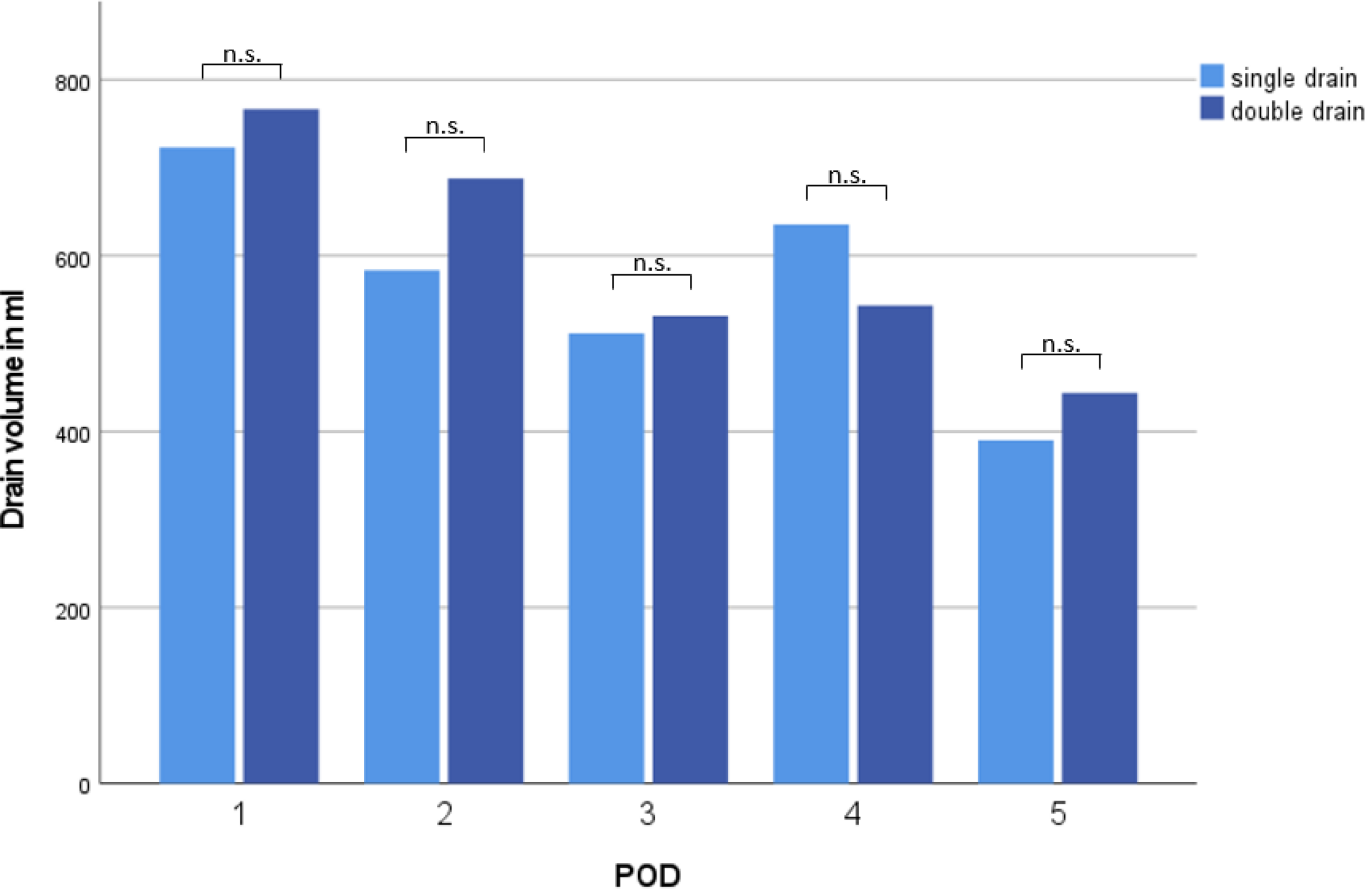
Chest drain volume POD 1–5.

Drain volume from POD 1–5 and total drain volume until removal of last chest drain were comparable between both groups (2,450 ± 1,511 ml vs. 2,809 ± 1,210 ml, *p* = 0.39; 3,200 ± 2,563 ml vs. 3,243 ± 15,923 ml, *p* = 0.41, Figure 2).

### Pulmonary complications, interventions, and diagnostics

After removal of the final chest drain, radiological diagnostics (chest x-ray and/or CT scan) showed right-sided pneumothorax in 5 (22.7%) patients in the single drain and 2 (9.1%) patients of the double drain group (*p* = 0.21, Table 3). In the single drain group, right-sided pneumothorax was clinically relevant Clavien–Dindo Classification (CDC) IIIa and required re-placement of chest drain in only 2 (9.1%) patients 0.43 days (±0.79) after chest drain removal, in the double drain group no patient required re-placement of chest drains (*p* = 0.30).

**Table 3:** Pulmonary diagnostics, complications, and interventions single drain.

	Single drain	Double drain	*p*-value
	*n* = 22	*n* = 22	
Pulmonary complications and interventions after removal of chest drains			
Total right	17 (77.3)	12 (54.5)	0.10^#^
Pneumothorax right	5 (22.7)	2 (9.1)	0.21^#^
Clinically relevant pneumothorax right (CDC > IIIa)	2 (9.1)	0 (0)	0.30^#^
Pleural effusion right	12 (54.5)	10 (45.5)	0.38^#^
Clinically relevant pleural effusion right (CDC > IIIa)	1 (4.5)	0 (0)	1.0^#^
Total left	13 (59.1)	19 (86.4)	0.04^#^
Pneumothorax left	0 (0)	2 (9.1)	0.244^#^
Clinically relevant pneumothorax left CDC IIIa	0 (0)	0 (0)	0.5^#^
Pleural effusion left	13 (59.1)	17 (77.3)	0.166^#^
Clinically relevant pleural effusion left CDC IIIa	0 (0)	0 (0)	–
Thoracic CT drainage and re-operations			
CT drainage thorax (Abscess with anastomotic leak)	1 (4.5)	0 (0)	1.0^#^
Re-operation thorax:	3 (13.6)	0 (0)	0.116^#^
Anastomotic leak (Re-thoracotomy with esophageal diversion)	1 (4.5)	0 (0)	
Chylothorax (retroperitoneal clipping of thoracic duct)	2 (9.1)	0 (0)	
Pulmonary diagnostics			
Number of postoperative chest radiographs	8.32 ± 3.46	7.86 ± 2.27	0.93*
Number of postoperative computed tomographies of chest and abdomen	0.73 ± 1.03	0.5 ± 0.86	0.40*

* T-Test/ Mann–Whitney-U.

^#^ Chi-square/Fisher’s exact test.

Although rate of right-sided pleural effusion after drain removal was high in both groups (*n* = 12, 54.5%, vs. *n* = 10, 45.5%, *p* = 0.38), only one patient in the single drain group had clinically relevant pleural effusion (CDC IIIa) and received interventional pleural catheterization 7 days after chest drain removal. No patient in the double drain group had clinically relevant pleural effusion (*p* = 1.0).

Double drain patients had a significantly higher overall number of left-sided pleural effusions/pneumothorax (19 (86.4%) vs. 13 (59.1%), *p* = 0.04) diagnosed by imaging after drain removal on the right side. Of these, no pneumothorax or pleural effusion was deemed to be clinically relevant and required re-chest drain in both groups.

The rate of thoracic CT drainages or re-operations did not differ between both groups (*n* = 1 (4.5%) vs. *n* = 0, *p* = 1.0; *n* = 3 (13.6%) vs. *n* = 0, *p* = 0.12).

No difference in the total number of postoperative chest radiographs (*p* = 0.93) or computed tomography of chest/abdomen (*p* = 0.40) could be detected between the single and the double drain group patients.

### Postoperative pain management

In both groups, the majority of patients had a perioperative epidural catheter [*n* = 17 (77.3%) vs. *n* = 14 (63.6%), *p* = 0.27, Supplementary Table S2].

Despite epidural catheter, additional pain medication was necessary for 15.4 ± 5.1 days in the single drain and 16.8 ± 7.3 days in the double drain group (*p* = 0.45).

Neither duration of epidural catheter nor total (6.8 ± 2.1 days vs. 6.6 ± 2.0 days, *p* = 0.7), oral (7.5 ± 4.9 days vs. 7.9 ± 4.4 days, *p* = 0.8) or intravenous (9.1 ± 5.3 days vs. 6.9 ± 2.7 days, *p* = 0.21) pain medication differed significantly between the two groups.

### Surgical complications, LOS, and mortality

Complications CDC> IIIa occurred in four patients (18.2%) in the single drain and in two patients (9.1%) in the double drain group (*p* = 0.66, Table 4).

**Table 4:** Surgical complications, LOS, mortality.

	Single drain	Double drain	*p*-value
	*n* = 22	*n* = 22	
Clavien–Dindo ≥ 3a	11 (50)	9 (40.9)	0.76^#^
Clavien–Dindo > 3a	4 (18.2)	2 (9.1)	0.66^#^
Anastomotic leak	2 (9.1)	1 (4.5)	0.4^#^
Pneumonia	1 (4.5)	1 (4.5)	1.0^#^
DGCE	5 (22.7)	1 (4.5)	0.09^#^
ICU stay (d)	4.18 ± 5.11	5.27 ± 3.45	0.01*
Hospital stay (d)	18.32 ± 6.24	17.95 ± 7.26	0.77*
30-d mortality	0	0	–
90-d mortality	1 (4.5)	0	0.31^#^

* T-test/ Mann–Whitney-U.

^#^ Chi-square/Fisher’s exact test.

Consequently, frequent perioperative complications such as anastomotic leakage, pneumonia, and Delayed gastric conduit emptying (DGE) were equally distributed between the single drain and double drain group (Table 4).

Intensive care unit (ICU) stay of single drain patients was significantly shorter with 4.2 ± 5.1 days vs. 5.3 ± 3.5 days (*p* = 0.01), LOS was comparable in both groups (18.3 ± 6.2 vs. 18.0 ± 7.3, *p* = 0.77). One patient died in the single drain group within 90 days and no death occurred in the double drain group (*p* = 0.31).

## Discussion

To our knowledge, this study is the first to compare the use of a single intercostal chest drain with double intercostal chest drains after robotic Ivor Lewis esophagectomy in a matched cohort of patients with EC.

Although time until removal of the final chest drain and ICU stay are significantly shorter in the single drain group, we could not demonstrate advantages in terms of overall and pulmonary complications, drainage-associated re-interventions, amount of diagnostic imaging, analgesic use, and LOS.

The later group of patients with one chest drain benefited from a surgical team with 2 years more experience in robotic esophageal surgery than the earlier group of patients with two chest drains, despite propensity matching.

Our study demonstrates that a single chest drain is at least not inferior to double chest drains in terms of pulmonary complications, re-interventions, or perioperative complications in the setting of an experienced surgical team.

On the management of chest drains after esophagectomy, solely Bull et al. published a systematic review in 2021 (18). Inclusion criteria were heterogeneous. Thus, studies were included that compared different types of chest drains, numbers, removal criteria, and routes of drains (intercostal vs. transhiatal) after both Ivor Lewis and McKeown esophagectomy (18). Twenty-seven studies [comprising two randomized controlled trials (RCTs)] with 2,564 patients were included. Three studies analyzed the number of chest drains after esophagectomy under different points of view (24–26): De Pasqual et al. and Tang et al. retrospectively reported their single-center experience with an additional anastomotic chest drain to a single thoracic drain for the timing of diagnosis and treatment of anastomotic leakage (24, 25). Both state that the additional anastomotic drain is of minor importance in diagnosis and treatment of anastomotic leakage. Data for drainage-associated pulmonary complications and re-interventions are not reported.

Asti et al. compared transhiatal with intercostal chest tubes after hybrid esophagectomy and also found a significant reduction in the use of analgesics (27).

Of greater interest for us, Cai et al. evaluated the need for a single chest drain (*n* = 32) vs. no chest drain (*n* = 18) after minimally invasive thoracoscopic esophagectomy (26). In contrast to our surgical method, surgery started with thoracoscopy and was afterward completed with laparoscopy and a cervical approach for anastomosis as McKeown esophagectomy. In line with our results, the incidence of postoperative complications was comparable, but the no chest drain group had lower postoperative pain scales. If this resulted in relevant benefits regarding perioperative pain medication or LOS, it was not reported.

Sato et al. analyzed the safety of early chest tube removal after McKeown esophagectomy with posterior mediastinal or retrosternal gastric conduit pull-up and cervical anastomosis in a matched cohort of 89 patients per group (28). Significantly more patients achieved first mobilization within 15 h postoperatively in the early removal group (89.8%) than in the late removal group (52%, *p* < 0.01). Multivariate analysis revealed that early chest tube removal was not a risk factor for pulmonary complications or thoracocentesis.

The limitations of this study are its retrospective study design, small sample size, and lack of data regarding postoperative mobilization. In addition, learning curve effects may have been relevant to a certain extent when comparing the two cohorts, since the medical team showed a 2 years’ experience with RAMIE in the single drain group time period. Furthermore, the study was performed in a single institution with a Caucasian patient cohort. Thus, our ﬁndings may not apply to other countries and ethnic groups. These data should be validated in a prospective multicentric setting.

To further optimize perioperative outcomes after RAMIE in the setting of ERAS protocols, innovative randomized, controlled trials regarding chest drain management are mandatory. Two aspects of interest to further address are timing of (28) and criteria for chest drain removal or even completely “drainless” RAMIE. Cai et al. already demonstrated that minimally invasive thoracoscopic esophagectomy without closed thoracic drainage is safe and feasible (26). Therefore, our research group has designed the RESPECT trial (NCT05553795) (29). The purpose of this randomized trial is to evaluate a very early removal of postoperative chest drains 3 h after Ivor Lewis RAMIE regarding postoperative pain, analgesic use, number of postoperative chest x-rays and CT scans, interventions, postoperative mobilization, postoperative morbidity, and mortality.

In conclusion, routine application of a single chest drain after RAMIE is safe and not inferior to double chest drains and does not seem to negatively affect perioperative outcomes in the setting of an experienced surgical team.

## Data Availability

The raw data supporting the conclusions of this article will be made available by the authors, without undue reservation.
