# Supplementary material for: Single chest drain is not inferior to double chest drain after robotic esophagectomy: a propensity score-matched analysis
Source: Front Surg. 2023 Jul 14;10:1213404. doi: 10.3389/fsurg.2023.1213404 (PMC10375402; doi:10.3389/fsurg.2023.1213404)
Supplement: Supplementary file 1 [file Table1.docx]

**Supplemental table 1:** Patient characteristics and histopathologic findings before PSM

|  | **Single drain** | **Double drain** | **p-value** |
| --- | --- | --- | --- |
|  | **n = 23** | **n = 51** |  |
| **Age (years)** | 61.78 (12.67) | 61.27 (7.68) | 0.83* |
| **Sex**  female  male | 4 (17.4)  19 (82.6) | 4 (7.8)  47 (92.2) | 0.22^#^ |
| **BMI (kg/m^2^)** | 27.1 (3.4) | 27.1 (4.9) | 0.98* |
| **ASA**  1  2  3  4 | 0  7 (30.4)  16 (69.6)  0 | 0  19 (37.3)  31 (60.8)  1 (2.0) | 0.65^#^ |
| **Charlson Comorbidity Index (CCI)** | 2,7 ± 0.47 | 2.65 ± 0.52 | 0.70* |
| **Histology**  adenocarcinoma  squamous cell carcinoma  others | 17 (73.9)  6 (26.1)  0 | 33 (64.7)  15 (29.4)  3 (5.9) | 0.45^#^ |
| **Neoadjuvant treatment**  chemotherapy  chemoradiation | 21 (91.3)  13 (56.5)  8 (34.8) | 41 (80.4)  21 (41.2)  20 (39.2) | 0.24^#^  0.42^#^  0.42^#^ |
| **pUICC stage**  1  2  3  4 | 12 (52.2)  2 (8.7)  7 (30.4)  2 (8.7) | 23 (45.1)  6 (11.8)  19 (37.3)  3 (5.9) | 0.87^#^ |

* T-Test/ Mann-Whitney-U

^#^ Chi-square/ Fisher´s Exact-Test
