# Supplementary material for: Single chest drain is not inferior to double chest drain after robotic esophagectomy: a propensity score-matched analysis
Source: Front Surg. 2023 Jul 14;10:1213404. doi: 10.3389/fsurg.2023.1213404 (PMC10375402; doi:10.3389/fsurg.2023.1213404)
Supplement: Supplementary file 2 [file Table2.docx]

**Supplemental table 2: Postoperative pain management**

|  | **Single drain** | **Double drain** | **p-value** |
| --- | --- | --- | --- |
|  | **n = 22** | **n = 22** |  |
| **Epidural catheter** | 17 (77.3) | 14 (63.6) | 0.27*# |
| **Duration of epidural catheter (d)** | 6.76 ± 2.14 6.57 ± 1.99 0.69^#^ |  |  |
| **Duration of any pain** **medication (d)** | 15.36 ± 5.05 | 16.82 ± 7.31 | 0.45^#^ |
| **Duration of oral pain** **medication (d)** | 7.5 ± 4.9 | 7.9 ± 4.4 | 0.8^#^ |
| **Duration of i.v. pain** **medication (d)** | 9.14 ± 5.26 | 6.86 ± 2.68 | 0.21^#^ |
| **Discharge with regular pain** **medication** | 8 (36.4) | 5 (22.7) | 0.32* |
| **Discharge with pain medication on demand** | 11 (50.0) | 10 (45.5) | 0.76* |

* T-Test/ Mann-Whitney-U

# Chi-square/ Fisher´s Exact-Test
