# Supplementary figures and images for: Single chest drain is not inferior to double chest drain after robotic esophagectomy: a propensity score-matched analysis
Source: Front Surg. 2023 Jul 14;10:1213404. doi: 10.3389/fsurg.2023.1213404 (PMC10375402; doi:10.3389/fsurg.2023.1213404)

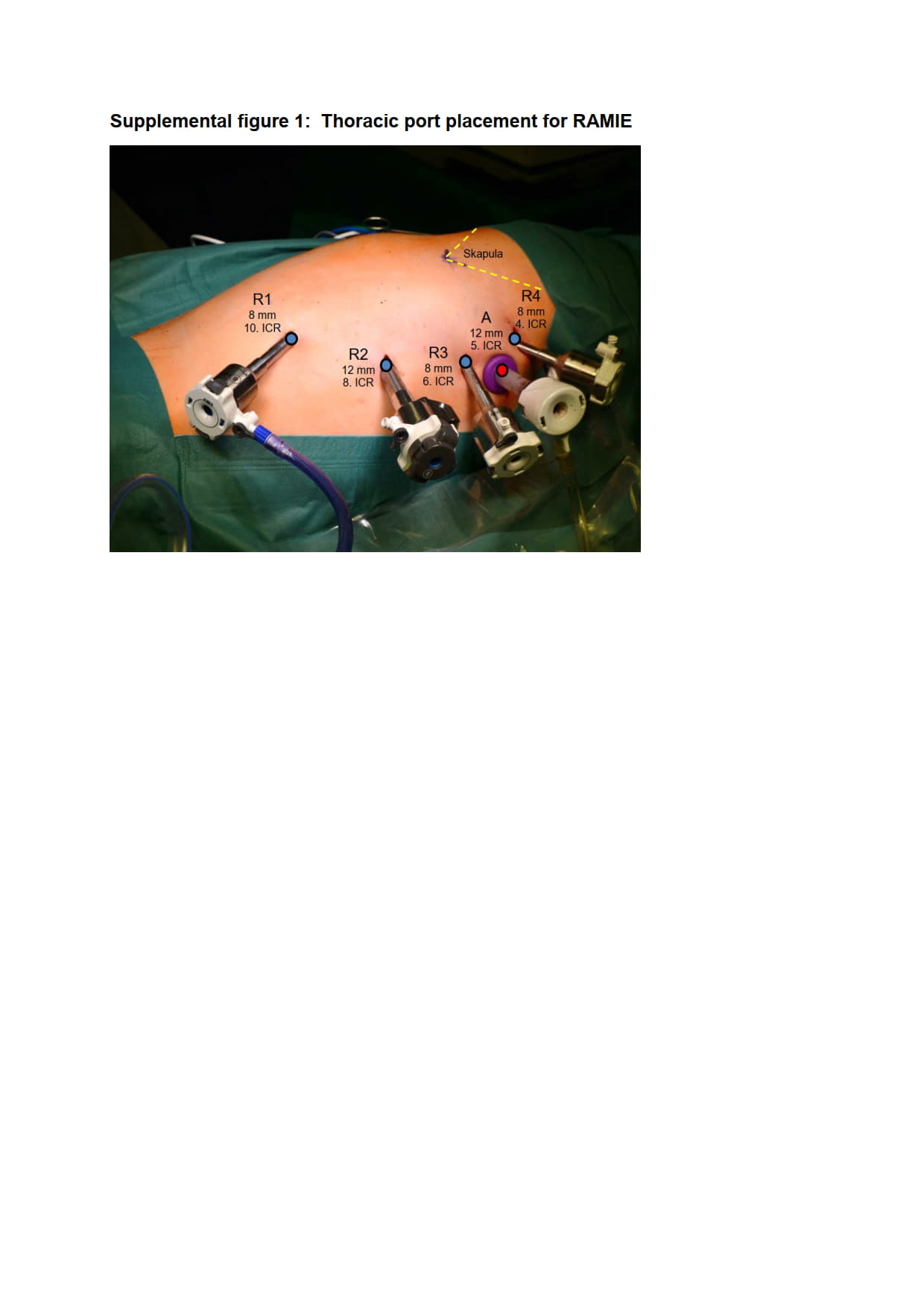

Supplement: Supplementary file 3 [file Image1.jpeg]

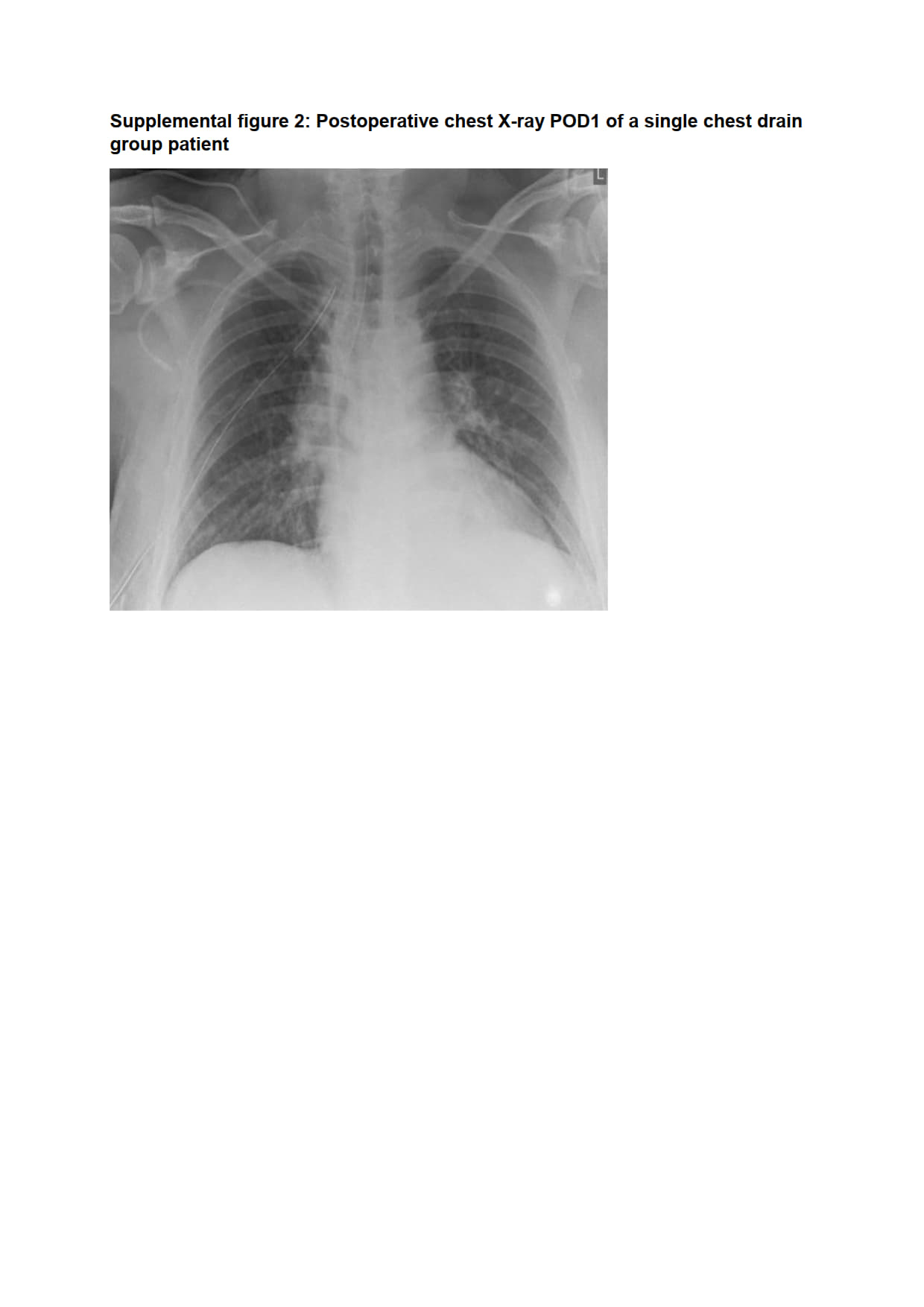

Supplement: Supplementary file 4 [file Image2.jpeg]
